# Disparities between Rural and Urban Areas of the Central Region of Saudi Arabia in the Utilization and Time-Centeredness of Emergency Medical Services

**DOI:** 10.3390/ijerph17217944

**Published:** 2020-10-29

**Authors:** Hassan N. Moafa, Sander Martijn Job van Kuijk, Dhafer M. Alqahtani, Mohammed E. Moukhyer, Harm R. Haak

**Affiliations:** 1 Department of Health Services Management, Faculty of Public Health and Tropical Medicine, Jazan University, Jazan 82817 2820, Saudi Arabia; 2 Department of Health Services Research, CAPHRI School for Public Health and Primary Care, Maastricht University, 6229 GT Maastricht, The Netherlands; H.Haak@mmc.nl; 3 Department of Clinical Epidemiology and Medical Technology Assessment, Maastricht University Medical Centre, 6202 AZ Maastricht, The Netherlands; sander.van.kuijk@mumc.nl; 4 Department of Quality Management, Saudi Red Crescent Authority, Ministry of Health, Riyadh 13251-8261, Saudi Arabia; mdhafer@srca.org.sa; 5 Department of Academic Development and Quality, Faculty of Applied Medical Sciences, Jazan University, Jazan 82511, Saudi Arabia; mmoukhyer@jazanu.edu.sa; 6 Department of Internal Medicine, Maxima Medisch Centre, 5631 BM Eindhoven, The Netherlands; 7 Department of Internal Medicine, Maastricht University Medical Centre, 6229 HX Maastricht, The Netherlands

**Keywords:** emergency medical services, utilization, response time, rural, urban

## Abstract

The purpose of this study was to explore differences in characteristics of missions dispatched by Emergency Medical Services (EMS) between rural and urban areas of Riyadh province in Saudi Arabia (SA). It also aimed at identifying weaknesses related to utilization and Response Time (RT). The study retrospectively evaluated 146,639 completed missions in 2018 by measuring the utilization rate in rural and urban areas. The study shows there are six times more ambulance crews available for rural areas compared to urban. There were 22.1 missions per 1000 urban inhabitants and 11.2 missions per 1000 in rural areas. The median RT for high urgent trauma cases was 20.2 min in rural compared to 15.2 min in urban areas (*p* < 0.001). In urban areas, the median RT for high urgent medical cases was 16.1 min, while it was 15.2 min for high urgent trauma cases. Around 62.3% of emergency cases in urban and 56.5% in rural areas were responded to within 20.00 min. Women utilized EMS less frequently. The RT was increased in urban areas compared to previous studies. The RT in the central region of SA has been identified as equal, or less than 20.00 min in 62.4% of all emergency cases. To further improve adherence to the 20′ target, reorganizing the lowest urgent cases in the rural areas seems necessary.

## 1. Introduction

Any health system in the world should have an integrated Emergency Medical Services (EMS) system in place to deal with public health emergencies such as out-of-hospital cardiac arrest (OHCA), stroke, and Road Traffic Accidents (RTAs). From the perspective of health equity, patient-centeredness, and time-centeredness, which are three out of six domains of healthcare quality declared by the Institute of Medicine [1], and people who are in urgent need of such services should be provided for regardless of their area, gender, age, ethnicity, or socioeconomic status [2]. Ambulances should be dispatched to them within an appropriate Response Time (RT), without any barriers limiting their accessibility.

Developing countries, such as those in the Arabian Gulf States (AGS), show a lower EMS utilization rate for acute coronary syndrome cases. For example, less than 25% of acute coronary syndrome patients in the AGS had been transported to the Emergency Department (ED) by EMS, while the rest were transported by private means [3,4]. A recent systematic review showed that most Saudi Arabian people (97.5%) in urban areas had used private transportation to the hospital for emergencies [5]. Another study in SA reported arrival modes for OHCA cases into EDs. It showed that EMS had transported two-third of OHCA cases caused by RTA, while the rest were transported by different means. As for other OHCA cases resulting from medical causes, only one-third of them were transported by EMS, while the rest were transported by other means [6]. Geographical location can play an important factor in the utilization and dispatching of an optimum resource [7,8,9,10,11]. Furthermore, RT varied between urban and rural areas as a result [12].

That same systematic review identified RT to be only been investigated in urban areas, especially in Riyadh, SA’s capital city, where it was found to be 10.2 min on average [13]. A more recent study reported that 81.9% of calls in Riyadh city were responded to within 25.0 min or less, and 65.8% were responded to in 15.00 min or less [14]. Nevertheless, no studies have investigated the demand nor RT in both rural and urban areas in SA based on different urgency levels.

The purpose of this study was to present an overview of the characteristic of EMS missions that were dispatched by different types of crew vehicles for different emergency and non-emergency types in urban and rural areas of the central region in SA, with a focus on RT and total EMS time, in order to identify weaknesses related to utilization and RT to be improved in the EMS system.

## 2. Materials and Methods 

### 2.1. Study Setting

This population-based registry study was conducted in the central region of SA, which can also be called Riyadh province [15]. It is home to about six million and seven hundred thousand people. This region has a geographical size of 404,240 km^2^ and is composed of 39 urban areas, defined as having a population of at least five thousand. Riyadh city is the capital city and has more than 5.2 million inhabitants. The rural areas are composed of about a hundred scattered villages located near or in-between urban areas and have fewer than five thousand people. The rural areas combined represent about half a million inhabitants (8.5% of the Riyadh province total population) [16].

**Figure 1:**
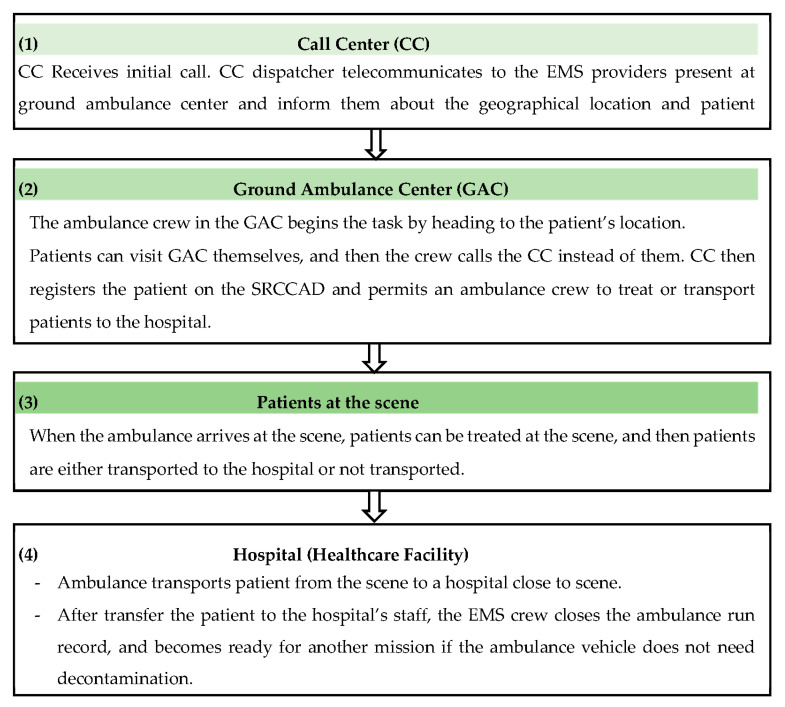
The Process Map of the Emergency Medical Services (EMS) Function in Riyadh Province for Every Single Ambulance Run.

The EMS in the central region is provided free of charge by the Saudi Red Crescent Authority (SRCA) through more than 100 Ground Ambulance Centers (GAC) distributed all over Riyadh province [17]. An ambulance is dispatched based on different urgency levels to the scene after the call had been triaged in the Call Center (CC) through using the algorithms and guidelines available in the Saudi Red Crescent Computer Aid Dispatching System (SRCCAD). The EMS operational process begins with the patient’s call with the CC, then the dispatching of the available closest crews to the patient at any time or place, and ending with the patient’s arrival at the hospital. This operational process is universally called the ambulance run. SRCCAD registers all ambulance runs to produce run reports over time. It shows the variation of performance for every individual emergency response. The EMS CC registers ambulance runs in the SRCCAD mainly by three means. The first registration is the most common and frequent: the CC registers the response to emergency calls from the callers in different geographical areas in the Riyadh province through a call-free number. The second registration occurs in the intermittent period in an unusual pattern through pre-planned official requests by the mega festivals and event organizers, but not through the CC’s call-free number. The third registration is limited to individual patients who go directly to the GAC due to their unawareness of the call-free number or their inability to call the CC. Likewise, this type of registration is also available for workers inside the GAC once one of the members is injured or becomes sick. In both cases, the GAC’s employees call the CC to inform them about the patient’s presence with them inside the center. The functional process is depicted in Figure 1.

The SRCCAD is programmed to display around the clock multiple time-interval indicators prepared in advance to monitor the crews’ performance closely. It has an information system backup that saves all ambulance runs. Therefore, the data of all EMS processes starting from patients’ calls and ending at the scene or with the patient’s arrival to healthcare facilities are recorded in the database to retrieve for auditing or quality improvement. This database serves as an operational information system to support the deployment of crews to the scene. It records some but not all demographic information, since the SRCA is an active member of the International Committee of the Red Crescent and the Red Cross. In turn, the CC members are required not to question their patients about their nationalities or races [18]. Nevertheless, the database also shows the patients’ geographical areas. More than this, it shows part of the triage. The retrieved times of all the different times intervals of the operational processes are also available in the database. This study employs all the records that had been entered into the database between January 2018 and December 31 of the same year.

### 2.2. Data Collection

The data were obtained through the operations and information department in the Riyadh branch directorate of SRCA. It is composed of all missions deployed to the scene in urban and rural areas in 2018 (1 January 2018–31 December 2018). The records were exported as an Excel file and saved in an encrypted file on a hard disc. 

The database includes clients’ demographic characteristics, geographical areas, the timeline of missions, reasons, types of emergency, and the outcomes of missions that ended with either non-conveyance or transportation to healthcare facilities. In the case of uncompleted missions, the reason was recorded. Table 1 defines each variable that has been used in this study.

### 2.3. Ethical Consideration

This study is part of a series of studies that were planned to be conducted. The study proposal was reviewed and approved by the Ethical Committee at Jazan University, Jazan, SA. Privacy and confidentiality were taken into consideration throughout the study. The ethical approval was issued under the following registry number: REC39/9-S085. 

### 2.4. Statistical Analysis

Data were exported from Microsoft Excel and converted to an IBM SPSS file (version 25) for further analyses. Characteristics of missions and emergency cases were described using counts and percentages (%). The aborted missions were excluded and the completed missions were considered and included in the analysis. The median RT with Interquartile Range (IQR) was computed separately for rural and urban areas, stratified by attributes such as the types of a medical emergency and urgency levels. Missions according to the emergency types that built in SRCCAD were clustered into six categories: medical emergencies, trauma emergencies, psychological emergencies, gynecological emergencies, non-emergency cases, and pre-planned missions (others). These categorizations were used to describe the data, but for the analysis four new clusters i.e., medical, trauma, others (psychological and gynecological), and non-emergency were used. The urgency levels of dispatching ambulances were divided by SRCCAD into three levels: high urgent, moderate urgent, and non-urgent levels. The cluster for emergency types was also stratified based on the three urgency levels. In addition, RT was compared to the benchmark of 20 min targeted by Saudi EMS. This benchmark was applied to all the missions, regardless of the urgency level. Differences between rural and urban areas in categorical characteristics were tested using Pearson’s chi-squared test. The independent samples t-test was used to compare RT between urban and rural areas.

## 3. Results

**Figure 2:**
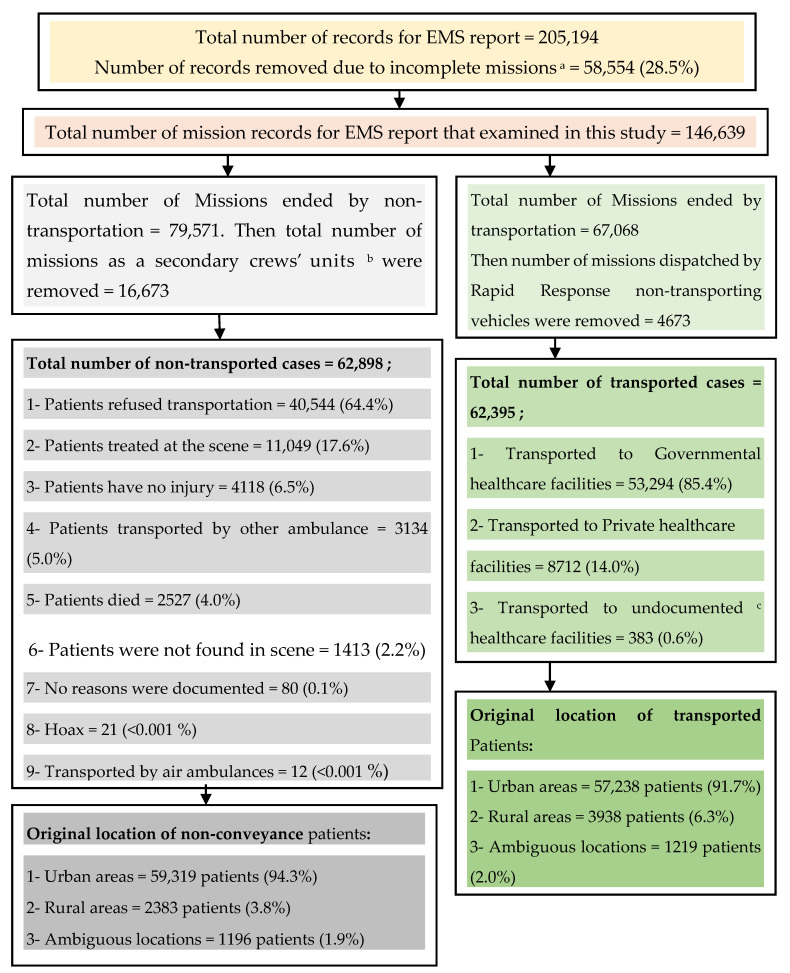
Flow Diagram for All missions in the Rural and Urban Areas of Riyadh Province, Saudi Arabia, during 2018 that either Transported the Patients to the Healthcare Facilities or Ended as a Non-conveyance Circumstances due to Multiple Reasons. ^a^ The decision made by the call center to stop the dispatched ambulance from continuing their mission toward the scene due to cancellation by a caller, wrong geographical location, false alarm, or nominate far crews to a patient. ^b^ Secondary crews are the qualified EMS personnel who dispatched to the scene and arrived afterward by another ambulance vehicle to support the first crews who arrived earlier. ^c^ Hospitals or primary care centers that were not documented by the Saudi Red Crescent database when the crews had arrived at them.

During the year 2018, the EMS information system recorded 205,194 records. Of these, 58,554 (28.5%) missions were categorized as non-completed or aborted missions due to one of the multiple common reasons that could happen to EMS systems, such as geographical misallocation, wrong assignment, and caller or patient not being found. There were 146,639 (71.5%) missions categorized as completed missions. In total, 96.5% of EMS completed missions were dispatched to the patients’ locations after patients called the CC. In comparison, 2.4% of those missions were not dispatched from the CC due to patients’ arrival themselves at the GACs. On the other hand, the preplanned ambulance missions that participated in community activities were represented by 1.1%. Therefore, those completed missions (146,639) are represented by the response to incidents or requests (*n* = 118,462; 80.8%). For 79.0% of patients related to these incidents or requests, only one vehicle had been dispatched; in 21.0% of cases, 2 or more vehicles had been dispatched. Ultimately, (*n* = 67,069; 45.7%) missions ended with patient transportation to a healthcare facility. The types of healthcare facility that patients have been transported to and the reasons for non-conveyance are depicted in Figure 2.

### 3.1. Utilization of Services in Rural and Urban Areas

Table 2 shows an overview of the absolute number of missions and the number of missions per 1000 inhabitants for all missions stratified by patient characteristics. Out of all the missions, the vast majority were for cases situated in urban areas (93.7%). The study shows that EMS missions for males were more than fir females, and non-elderly adults mostly requested the EMS. The available ambulance crews for rural and urban areas were 25 and 4 per 100,000 people a year, respectively.

EMS utilization differences between rural (11.2 mission per 1000 inhabitants per year) and urban areas (22.1 missions per 1000 inhabitants per year) were found in SA’s central region. In addition, the sex distribution differed significantly between rural and urban areas. Although the percentage of missions performed for males was similar, more missions were performed for patients of unknown sex (32.7%) in rural areas, whereas more missions for female patients (30.1%) were seen in urban areas. The majority of emergency missions were performed during the post-working period (75.2%) compared to working hours (24.8%). Although there were statistically significant differences between rural and urban areas, this difference was not clinically meaningful. Most patients in urban areas demanded EMS for medical emergencies (40.3%), whereas the patients in rural areas demanded them mostly for traumatic emergency (54.0%, (*p* < 0.001)). This study reveals that 67.8% of incoming calls to CC from rural areas were due to high urgent emergency cases, while in urban areas, the percentage of calls for the high urgent category was 50.8%, (*p* < 0.001). The majority of patients in urban areas (53.8%) were not transported after EMS crews had contacted them at the scene, and EMS transported less than half (46.2%) to healthcare facilities. In contrast, in rural areas, patients were most often transported to healthcare facilities ((60.3%), (*p* < 0.001), see Table 3).

### 3.2. Response Time in Rural and Urban Areas

This current study shows that the overall median RT differed between urban and rural areas (17.0 min (IQR: 11.8–23.9) for urban compared to 17.6 min (IQR: 9.8–28.6) for rural, (*p* < 0.001)). When stratifying by emergency types based on the three different levels of urgency, we found that the median RT was significantly different between urban 15.2 min (IQR: 10.7–21.7) and rural 20.2 min (IQR: 12.9–30.9) areas (*p* < 0.001) for the highest urgency traumatic emergencies. For the highest urgency level in both areas, the cases resulting from traumatic causes in urban areas were the shortest in median RT compared to the rest. In contrast, in rural areas, the cases resulting in medical causes were the shortest. The median RT for the non-emergency cases in rural areas (13.6 min, IQR: 3.5–24.9) was found significantly shorter than in urban areas (20.0 min, IQR: 13.6–28.4, (*p* < 0.001. See Table 4). 

The study also reveals that the RT for 62.3% of emergency cases in urban areas and 56.50% in rural areas, was equal to or less than 20.00 min. For the highest urgent traumatic cases in rural areas, the EMS responded to 49.90% of them in equal or less than 20.00 min. For the lowest urgent non-emergency cases in urban areas, the result shows that 49.9% of cases were responded to in 20.00 min or less (see Table 5).

### 3.3. Total EMS Time in Urban and Rural 

In total, 61,129 patients had been transported to healthcare facilities. The overall median total EMS time for the highest urgency cases differed between urban and rural areas, 77.5 min, (IQR: 61.0–95.5) for urban compared to 79.1 min, (IQR: 58.2–109.2) for rural (*p* < 0.001). After stratifying by emergence type for the highest urgency level, the median of total EMS time was found to be significantly different between urban (79.7 min, (IQR: 63.7–97.1)) and rural (69.1 min, (IQR: 50.2–94.6)) areas (*p* < 0.001) for medical emergencies. For a traumatic emergency, the median total EMS time for urban was 74.1 min, (IQR: 58.0–92.0), and was 82.0 min, (IQR: 60.4–112.7) for rural areas, (*p* < 0.001). 

## 4. Discussion

This study’s main goal was to explore the characteristics of EMS missions that had been dispatched for out-of-hospital emergency purposes in both urban and rural areas in the central region in SA. The systematic review by Moafa H.N et al. found that no EMS research in rural areas of the AGS, including SA, had been performed yet [5]. Most previous EMS studies that investigated the utilization or EMS time-centeredness in the central (Riyadh) region focused on the capital, Riyadh city [6,13,20]. The overall direction of results from our study showed remarkable differences in utilization and time-centeredness between urban and rural areas. 

Despite the EMS services being free of charge and easy to access, we found a substantial variation of services demand in urban areas compared to rural areas. For example, our study shows that there were 22.1 missions per 1000 urban inhabitants and 11.2 missions per 1000 in rural areas (Table 2). The possibility of unmet needs cannot fully explain the EMS’s low utilization by people living in rural areas. This justification is evidenced by the absolute number of available EMS crews in rural areas. However, no previous SA studies have investigated the different means of transportation to ED in rural areas [5]. In the United States, there were significant disparities in the type of emergency cases besides RT due to inequality between urban and rural areas [9,21,22]. In Australia, Buck Reed et al. compared the mode of transportation for three geographical areas. They found that 11.9% of rural Australians had ever used an ambulance compared to 26.6% of urban people [23]. In our study, despite lower demand by people in rural areas, most calls were for support for high urgent cases (Table 3). This finding may have caused the higher transportation rate to health care facilities in rural (60.3%) compared to urban areas (42.6%), which is similar to the results of a study from Germany, which found that emergency transport rates from rural areas were higher (82.8%) than in sizeable urban areas (68.6%) for all urgent categories [24]. 

The elderly were the most frequent EMS users in urban and rural areas with 174.4 cases per 1000 a year, while children were the least frequent users (2.8 per 1000 a year) (Table 2). This difference is similar to what has been found in the United States [10,25,26]. However, Saudi children utilize EMS far less than American children (26 per 1000 a year) [27]. Saudi females were less frequent EMS users in urban and rural areas, with 15.3 cases per 1000 a year, although we did see an increase in unknown gender (Table 2). Exposure of women to high-risk jobs and RTA is very low for many cultural reasons. For example, women were not allowed to drive motor vehicles in 2018. Another Saudi study found a 1 to 9 ratio of women compared to men for RTA admissions to hospitals [28]. This finding is in agreement with a recent systematic review that showed that women in the AGS were less likely to use EMS compared to other means of transportation when they arrived at the ED [5]. 

During this study, the predetermined targeted RT for the EMS in Riyadh province was 20.00 min. This study reveals that the median RT to high urgent cases was longer in rural than in urban areas. On the contrary, the median RT to moderate and non-emergency cases was shorter in rural than in urban areas (Table 4). However, we found that the median RT in rural areas was longer than for urban for all emergency cases (Table 4). This finding is often the case, as shown in a recent review [29]. Our finding of RT was almost double that found in the United States [12,30]. Despite the optimum coverage of ambulance centers in rural areas (25.9 crews/100,000 inhabitants), which was six times more than in urban (4 crews/100,000 inhabitants), this study has shown that the median RT in rural areas for high urgent trauma cases is the longest in period (20.2 min) compared to other emergency cases in Riyadh province. This could, in part, be due to mass gatherings and traffic jams in highways, which curb the ambulances crews’ access to the scene and sometimes may have become a barrier for crews to reach and treat the injured people properly [31]. Patients’ access to EMS through visiting the GACs without calling CC is quite common in rural areas, especially with non-emergency cases (*n* = 335; 25.0%) and moderate urgency level cases (*n* = 85; 13.0%). Therefore, this may explain the shorter median RT time for non or moderate urgent cases than high urgent cases in the rural setting.

In this study, the RT (*n* = 24,557; 67.6%) of high urgent medical cases was less than 20.00 min in all areas (Table 5). In SA, the RT benchmark of 20.00 min is longer than in developed countries [12,32]. In our opinion, an RT of 20.00 min in SRRCAD is too long to respond to cases such as OHCA [33]. The global challenge nowadays is to respond to OHCA as soon as possible [19,34]. A study conducted by Alnemer K et al. found that the average RT for OHCA in Riyadh city was 13.2 min (standard deviation 7.9 min) [20]. 

The coverage of GACs in urban areas is almost twice that of other Asian countries such as South Korea and Singapore. Yet, these countries have shorter RT than we observed in Riyadh [35]. One possible reason for this is that they do not dispatch ambulances for non-emergency cases. ^33^ Likewise, the National Health Security in the United Kingdom has provided all patients with a clear health policy on their website, stipulating that EMS does not respond to non-emergency cases. They have instructed them to use other means specified for such cases [36,37]. We found that 29.9% of dispatched ambulances on the scene in urban areas were for non-emergency cases (Table 3). Such a procedure adds an overload to the EMS systems and delays other life-threatening case responses and may lead to excess mortality [38,39]. 

Our results are considered the first population-based study in the Arabian Gulf States for one whole year of all EMS missions in a large area, rural and urban in SA including the capital city of the country. It encompasses a large database, able to present the differences between rural and urban, and the differences between different types of missions. Although not generalizable, it is of use for implementing an improvement in the system in SA and other regions or countries, applying a similar way of access to EMS.

One limitation of this study was that the database used was designed for mainly operational purposes and to some extent for academic purposes. It could not be linked to follow-up records from the hospitals to allow for the evaluation of patient outcomes. Another limitation was that gender was missing for a number of cases. Although we understand the difficulties in identifying the patient’s gender at the time a call is placed for certain major incidents such as car accidents, it cannot explain the large differences in the proportion of unknown gender between urban and rural areas. In addition, in SA, EMS can be obtained by two means: by calling the EMS CC and by attending to a GAC. Although direct visits to GACs are unique to SA, call-in times could be compared across countries for future studies on the condition of not including the data of visiting GACs. However, the RT and total EMS time in this study are not comparable to other international EMS systems that only include missions for patients that called. Services in rural areas can be improved by conducting further studies into the variation of service utilization between genders of different ages and whether the gender and cultural factors can affect the outcome of EMS periods. Moreover, this study might support researchers who intended to develop further studies to determine the barriers that curb people from communicating with the CC and lead them to visit the GACs instead. Further studies focusing on the seasonal variation role in EMS utilization and RT is warranted since this study did not investigate this attribute.

## 5. Conclusions

In conclusion, this study shows that the EMS utilization in rural areas was lower compared to urban areas, specifically in terms of medical emergencies. Women were less frequent EMS utilizers in the Riyadh province, possibly because of a lower number involved in hard, risky jobs and that are less vulnerable to RTA, because of not driving cars. RT seems to increase in urban areas compared to previous studies. Compared to the Saudi EMS benchmark of 20.00 min, around 62.3% of cases in urban areas and 56.5% in rural areas had a RT shorter than 20.00 min. To further improve adherence to the 20-min target, the reorganization of lower urgency cases in rural areas seems necessary. Furthermore, updating the geographical distribution of EMS dispatch centers might be necessary.

## Figures and Tables

**Table 1:** Definitions and Abbreviations.

Category	Terminology/Abbreviation	Definition
Geographical areas	Rural areas	Areas that have a total population of fewer than 5000 inhabitants or an area outside the categorized urban areas.
	Urban areas	Areas where metropolitan, and micropolitan cities are located and that have a total population of equal or more than 5000 inhabitants. An example of the largest urban area is Riyadh, the capital of Saudi Arabia, and it has a total population of 5,271,991.
	Ambiguous	Areas that could not be categorized into rural or urban (e.g., on a highway) or a combination of unavailable or invalid data in the database.
Age category	Child	Patients with an age below 15 years.
	Adult	Patients equal to, or over 15 years, but younger than 60 years old.
	Elderly	Patient over 60 years of age.
Types of Emergency cases	Medical emergency	Sudden medical emergency illness of any type requiring immediate intervention because human physiology was severely affected. Examples are chest pain, bronchial asthma, coma, and Out-of-Hospital Cardiac Arrest.
	Traumatic emergency	Sudden severe emergency injury of any type caused by blunt or sharp objects such as Road Traffic Accident (RTA), falling from a high building, and work injury.
	Psychological emergency	Acute and sudden disturbance of patient’s behavior and attitude, which if not treated soon, could result in patients harming themselves, family, or the community surrounding them.
	Gynecological emergency	Sudden condition relating to the female reproductive system that affects the woman’s lives, such as abortion, vaginal bleeding, or complications during childbirth.
	Prehospital non-emergency	Minor medical illness or injury that occurred without disturbing vital signs and does not need immediate intervention by prehospital EMS providers and can be treated by the general practitioners, such as seasonal flu and tensional headache.
SRCA levels of urgency	High urgent cases	Any life- threatening emergency calls such as cardiac arrest, severe traumatic injuries, or cerebral stroke that requires immediate advanced life support intervention and is given the highest priority for the crews to be dispatched for them by SRCCAD in the CC.
	Medium urgent cases	A group of medical and other illnesses and traumatic injuries such as febrile convulsion, psychological attack, alcoholism, uncomplicated diabetic prioritized by SRCCAD and take the second priority if the life-threatening cases calls come together at the same moment.
	Low/non-urgent cases	A group of mild medical illnesses and minor injuries such as the common cold, mild muscular pain that does not affect the human body physiology and therefore, does not require immediate medical intervention, which in turn can be categorized by SRCCAD as a tertiary priority.
Time period of emergency medical services	Response time	The time elapsed starting from receiving a call in the CC and ended by the arrival of the ambulance’s crew to the scene.
	Total EMS time	The total time measured starting from receiving the call in the CC and ending with the ambulance handover of the patient to the emergency department staff in the hospital.
Outcome of emergency service missions	Completed missions	The mission that is activated by the CC when the emergency services are requested by the emergency caller, and then the EMS crew arrived at the caller address and ended either by non-conveyance or transportation to the healthcare facility.
	Types of completed missions	1- Non-conveyance missions: the condition where the ambulances arrived at the scene and the patients after having been examined or treated on the scene, transport to the health facility is not necessary or is refused by the patient. 2- Transported mission: as an ambulance transported the patient from the scene to the hospitals’ emergency department.
	Aborted missions	The decision that made by CC to stop the dispatched ambulance crew from continuing their mission toward scene due to reasons such as cancellation by a caller, wrong geographical areas, false alarm or the nominated crews being far from the patient.
Missions period	Working time	The time that starts from 8:00 AM to 4:00 PM from Sunday to Thursday.
	Rest time	The time that starts from 4:01 PM on the same day up to next day 7:59 AM side by side with 48. 00 h of the weekend Friday and Saturday.
Crew dispatched at the scene	Crews dispatched to urban areas	Two professional EMS staff who dispatched from urban GAC by one of the following vehicles: Mobile Intensive Care Unit, Non-transporting Fast Responding Vehicle, or Ambulance type II to urban areas and might rarely participate in close rural areas if the SRCCAD nominated them due to a shortage of staff in the rural area.
	Crews dispatched to rural areas	Two professional EMS staff dispatched from rural GAC by Ambulance type II to rural areas and might rarely participate in close urban areas if the SRCCAD nominated them due a shortage of staff in the urban area.
EMS crew’s arrival to scene	Primary crews	The qualified EMS personnel who were dispatched by the dispatch department in the CC to first arrive by any vehicle and contacting the patient upon arrival at the scene.
	Secondary crews	The qualified EMS personnel who were dispatched to the scene and arrived afterwards by another ambulance vehicle to support the first crews that arrived earlier.
Health care facility	Governmental	Non-profit healthcare services that are provided and funded by the Saudi government through the ministry of health, university hospitals, military hospitals, and security hospitals, and national guard hospitals for all Saudi citizens.
	Private	Hospitals, or primary healthcare centers that are not free-of-charge and are operated by non-governmental healthcare firms.
Call center (CC)	The workforce consisting of different office disks operated by professional staff who are able to communicate with the emergency caller and are also able to operate software of SRCCAD and the telecommunication apparatus.	
Ground Ambulance Center (GAC)	The EMS facilities including all structural logistic elements such vehicles, medical equipment, telecommunication machines, and EMS providers.	
List of abbreviation and definition	OHCA	Out of hospital cardiac arrest: stopping of cardiac pulse activity, confirmed by the absence of signs of circulation outside the hospital field [19].
	RTA	Road Traffic Accident is an accident that occurs on the road without prior planning by any one of the involved parties and leads to death, or temporary or permanent disability.
	SRCA	The Saudi Red Crescent Authority, which is considered to be the main EMS provider for prehospital emergency healthcare in Saudi Arabia.
	SRCCAD	The out-of-hospital information system, designed by Saudi programmers, which includes all relative structural information needed to connect all resources in order to respond properly to patients with different emergency types linked to already designated priorities.

**Table 2:** Description of the Total Missions Dispatched by Sex, Age category and Geographical Area.

Category	Categorization	Mission (*n* = 146,639)	Population (*n* = 6,792,776) (100%)	N of Calls’ Missions per 1000
Missions	All Missions	146,639 (100%)	6,792,776 (100%)	21.6
	Transported	67,068 (45.7%)	6,792,776 (100%)	9.9
	Non-conveyance	79,571 (54.3%)	6,792,776 (100%)	11.7
Sex	Male	83,702 (57.1%)	3,995,352 (58.8%)	21.0
	Female	42,893 (29.3%)	2,797,424 (41.2%)	15.3
	Unknown ^a^	20,044 (13.7%)	NA	NA
Age category ^b^	Child (<15 years.)	5034 (3.4%)	1,782,648 (26.2%)	2.8
	Non-elderly adult (15–59)	73,528 (50.1%)	4,794,176 (70.5%)	15.3
	Elderly (≥60 years)	37,662 (25.7%)	215,952 (3%)	174.4
	Unknown ^a^	30,415 (20.7%)	NA	NA
Call geographical areas ^c^	Urban	137,347 (93.7%)	6,213,184 (91.5%)	22.1
	Rural	6487 (4.4%)	579,592 (8.5%)	11.2
	Ambiguous	2805 (1.9%)	NA	NA

^a^ Combination of unavailable or invalid data for reasons such as mass causalities or poor reporting. ^b^ The determination of age groups is based on the information provided in the Saudi Statistical Authority report of 2010. ^c^ The areas are categorized by the Saudi Statistical Authority report of 2010.

**Table 3:** Describing the Differences of Missions Utilized per Patients in Categorical Variables Between Geographical Area Groups ^a^.

Category		Geographical Areas			
		Urban n (%)	Rural n (%)	Total n (%)	*p*-Value
Sex	Male	62,511 (56.7)	3405 (56.7)	65,916 (56.7)	
	Female	33,124 (30.1)	638 (10.6)	33,762 (29.1)	<0.001
	Unknown ^b^	14,551 (13.2)	1960 (32.7)	16,511 (13.7)	
Age category	Child (<15 years.)	3715 (4.2)	155 (5.0)	3870 (4.2)	
	Non-elderly adult (15–60)	55,635 (62.5)	2330 (75.3)	57,965 (62.9)	<0.001
	Elderly (≥60 years.)	29,708 (33.4)	609 (19.7)	30,317 (32.9)	
Mission period	Rest Time	82,590 (75.0)	4592 (76.5)	87,182 (75.2)	<0.001
	Working Time	27,596 (25.0)	1411 (23.5)	29,007 (24.8)	
Emergency reasons for calls	Medical Emergency	44,404 (40.3)	1361 (22.7)	45,765 (39.4)	
	Trauma	29,290 (26.6)	3244 (54.0)	32,534 (28.0)	
	Psycho-psychiatric Emergency	929 (0.8)	9 (0.1)	938 (0.8)	<0.001
	Gynecological Emergency	1171 (1.1)	18 (0.3)	1206 (1.0)	
	Non-emergency	32,943 (29.9)	1302 (21.7)	34,245 (29.5)	
	Others ^c^	1449 (1.3)	69 (1.1)	1518 (1.3)	
Urgency levels	High Urgency Level Cases	55,967 (50.8)	4068 (67.8)	60,035 (51.7)	
	Medium Urgency Level Cases	19,677 (17.9)	553 (9.2)	20,230 (17.4)	<0.001
	Low Urgency Level Cases	34,542 (31.3)	1382 (23.0)	35,924 (30.9)	
Mission outcome	Non-conveyance	59,319 (53.8)	2383 (39.7)	61,702 (53.1)	<0.001
	Transported to Healthcare Facility	50,867 (46.2)	3620 (60.3)	54,487 (46.9)	

^a^ There are limitations in identifying patient numbers for a limited number of missions such as missions dispatched for major casualty incidents; therefore, the report number for such missions are considered as a single patient. ^b^ Unknown sex could be related to an incident for multiple patients in one casualty incident or could be poorly reported by staff; therefore, sex category was not documented. ^c^ An ambulance was dispatched to the scene as preplanned for a festival or an event without a direct call to an emergency call-free number.

**Table 4:** Describing Median Response Time for Cases Located in Urban and Rural Areas Based on Triaging of SRRCAD of Three Types of Urgency Levels and Three Different Causes of Calls.

Urgency Levels of EMS Missions		Urban	Rural	*p*-Value
All Missions	Number of cases	108,732	5934	
	Median (IQR)	17.0 (11.8,23.9)	17.6 (9.8,28.6)	<0.001
High urgent (overall)	Number of cases	54,726	3983	
	Median (IQR)	15.8 (11.2,21.8)	19.0 (11.7,30.0)	<0.001
High urgent medical	Number of cases	35,172	1138	
	Median (IQR)	16.1 (11.5,22.2)	16.2 (8.6,26.7)	<0.001
High urgent trauma	Number of cases	19,074	2840	
	Median (IQR)	15.2 (10.7,21.7)	20.2 (12.9,30.9)	<0.001
High urgent for others ^a^	Number of cases	480	5	
	Median (IQR)	17.0 (12.8,23.0)	16.9 (6.2,25.5)	0.700
Moderate urgent (overall)	Number of cases	21,032	649	
	Median (IQR)	16.38 (11.7,22.6)	15.43 (8.8,25.4)	<0.018
Moderate urgent medical	Number of cases	9196	223	
	Median (IQR)	15.91 (11.56,21.7)	15.95 (10.1,28.2)	<0.001
Moderate urgent trauma	Number of cases	10,216	404	
	Median (IQR)	16.53 (11.7,22.9)	15.38 (7.0,24.8)	<0.001
Moderate urgent for others ^b^	Number of cases	1620	22	
	Median (IQR)	18.4 (13.0,25.8)	10.65 (8.6,21.6)	0.470
Low non-emergency cases	Number of cases	32,974	1302	
	Median (IQR)	20.0 (13.6,28.4)	13.6 (3.5,24.9)	<0.001

^a^ Dispatched missions for calls for gynecological and psychological reasons and categorized by SRCCAD as being at a highly urgent level. ^b^ Dispatched missions for calls resulting in gynecological and psychological reasons and categorized by SRCCAD as being at a moderately urgent level.

**Table 5:** Percentage of Response Times in 20 min or Less Based on Urgency Levels.

Urgency Levels of EMS Cases	All Locations n (%)	Urban n (%)	Rural n (%)	*p*-Value
Overall EMS cases	71,571 (62.4)	68,680 (62.3)	3391 (56.5)	<0.001
Highly urgent medical emergency	24,557 (67.6)	23,861 (67.8)	696 (61.2)	<0.001
Highly urgent traumatic emergency	15,001 (68.5)	13,604 (71.3)	1397 (49.2)	<0.001
Moderately urgent medical emergency	6506 (69.1)	6363 (69.2)	143 (64.1)	0.110
Moderately urgent traumatic emergency	6946 (65.4)	6689 (65.5)	257 (63.6)	0.440
Low non-emergency cases	17,284 (50.5)	16,432 (49.9)	852 (65.4)	<0.001
